# A Key to Genome Maze in 3D

**DOI:** 10.1016/j.gpb.2016.02.002

**Published:** 2016-02-11

**Authors:** Zhihua Zhang

**Affiliations:** CAS Key Laboratory of Genome Sciences and Information, Beijing Institute of Genomics, Chinese Academy of Sciences, Beijing 100101, China

How a genome with linear length over meters is compacted into the micrometer-sized nucleus of higher eukaryotic cells, and how this compaction affects and is affected by the genome functionalities have puzzled biologists for decades. There are mainly two classes of technologies that are dedicated to the probing of genome spatial organization. Image-based analyses of three-dimensional (3D) fluorescence *in situ* hybridization (FISH) [1] have been widely used in genome spatial research. This technology family has contributed to many early landmark discoveries of 3D genome (*e.g.*, finding that chromosomes occupy distinct non-overlapping territories in interphase [2]), and continues to assist biologists in zooming into the genome spatial structure [3]. The other technology family is 3D genome mapping, which takes advantage of the next-generation sequencing (NGS) technology to sample the proximity ligated genome fragments [4], [5] as chromatin interactions. Chromatin interaction analysis by paired-end tag sequencing (ChIA-PET) and high-throughput chromosome conformation capture (Hi-C) are two representative technologies in this class [4], [5]. ChIA-PET detects chromatin interactions mediated by specific protein factors, thus it is more specific and has higher resolution in comparison to Hi-C, which captures all chromatin contacts comprehensively. Since the introduction of ChIA-PET and Hi-C seven years ago [6], [7], more detailed spatial genome architectures have been revealed, such as topologically associated domains (TAD) [8] and clustered gene promoters for transcription [9]. However, both Hi-C and ChIA-PET technologies face challenges including data noise stemming from complicated experimental protocols, exponential explosion in the sequencing depth required for higher resolution analysis, and large starting cell numbers. Some endeavors have been made to address one or some of such challenges. For instance, DNase I or micrococcal nuclease have been used to substitute restriction enzymes for chromatin fragmentation, enabling higher resolution in chromatin contact mapping [10], [11].

A recent paper published by Tang et al. in the last issue of *Cell* in 2015 represents a substantial progress in addressing the aforementioned challenges [12]. The senior author Yijun Ruan is the pioneer developer of ChIA-PET technology. In this article, they reported a new version of ChIA-PET aiming to reduce the inter-complex ligation, which is the major source of experimental noise, through two innovative modifications to the original protocol. Instead of using two half-linkers with a sticky end, the proximity ligation in the new protocol is carried out using a single bridge-linker with a 3′-dT overhang to enrich the intra-complex ligations. Moreover, by employing the Tn5 transposase-mediated fragmentation, the authors are able to produce longer sequence tags (2 × 150 bp *vs.* 18 bp in the previous version), thus resulting in higher mapping confidence and base pair coverage.

The authors applied this long read ChIA-PET to four human cell lines using CCCTC-binding zinc finger protein (CTCF) and RNA Polymerase II (RNAPII) as the target proteins for immunoprecipitation. CTCF is a transcription factor that has been identified as one of the key architectural proteins in mammalian 3D genome organization [13]. Since CTCF recognizes and binds to an asymmetric DNA motif with core sequence CCCTC [13], CTCF binding is usually considered to be directional. In this newly-generated CTCF-mediated chromatin contact map, a surprisingly high portion of interactions were found to be so called “tandem loops”, which are mediated by CTCF dimers with the motifs facing the same direction in the genome. While previous Hi-C data [14] and detailed functional analysis [15] suggested that “convergent loop”, *i.e.*, the two CTCF binding motifs are facing each other, dominates the genome. In the paper, Tang et al. argued that this newly-discovered tandem loops might represent the power of specific enrichment in CTCF ChIA-PET experiments. This argument was further supported by the consistent binding pattern of cohesin, another key structural mediator protein in mammalians. Based on these observations, the authors proposed hairpin loop and coiled loop models for convergent and tandem looping, respectively. The two different looping types proposed may be important for cells to modularize the spatial organization of genome, because the convergent loops, which define the so-called CTCF-mediated chromatin contact domains (CCDs), are more likely to be found between distal genome loci, and largely consistent with previously-reported TADs, whereas tandem loops are evenly distributed within CCDs. Authors did not further discuss the thermodynamical difference between the two proposed loop types, which I believe can be an interesting topic for the theoretical biophysics community. Nonetheless, multiple -omics analyses support the plausible significance as the two looping types have remarkably different pattern in determining the tissue-specific *vs.* housekeeping expression of genes. These data indicate for the first time that the CTCF/cohesin-mediated chromosome structures and the spatial organization of gene transcription are highly cooperative.

The next question the authors tried to address with this newly-generated high-resolution data is whether a chromatin loop change can really affect gene expression, or even more, have visible phenotype effects. The authors adopted a smart strategy in tackling this issue. They noticed that a cell type (GM12878) in the dataset has well-phased single nucleotide polymorphisms (SNPs), thus a large portion of loops can be mapped to its parental originations. As a result, they identified nearly 300 haplotype-biased CTCF loop anchors and about 2000 CTCF loops, as well as similar number of RNAPII anchors and loops. The first striking finding is that a single SNP could directly alter chromatin topology and function at a fine scale, at least alter an individual loop. The authors identified a recurrent heterozygous variation among the CTCF core binding motifs at position 14, which has been previously reported as a key site in CTCF functionality [16], [17]. The nucleotide change at the site between two alleles fully explains the looping structure at the loci in GM12878, beautifully demonstrating the critical effect of CTCF binding in the formation of the chromatin looping. Furthermore, the authors systematically assessed disease association of the disrupted CTCF-mediated chromatin contacts by known disease-associated SNPs. Indeed, strong association between the two can be found in this new dataset, highlighting the potential applications of allelic chromatin contact mapping for identification of causal disease-associated SNPs.

Finally, the authors modeled the physical 3D structure of human genome by simulations with combined CTCF and RNAPII ChIA-PET data. In addition to well-known chromosomal structure features, such as the chromosomal territory, the model supports an emerging general feature of chromosome topology. That is, even in interphase with much less condensed condition in general, chromosomes always maintain a core axis, which is supposed to be much more condensed heterochromatin segments, like a lampbrush chromosome. The authors suggested that this lampbrush structure explains not only how a chromosome maintains its large-scale physical properties, *e.g.*, the chromosomal territory, but also the separation of repressive and active compartments in one chromosome as detected in Hi-C data [7]. To separate the two compartments, CTCF and cohesin are supposed to bind simultaneously to the surface of the core axis, forming an interface between the condensed inner heterochromatin and the outer loose domains. Imaging analysis indeed observed the spatial overlap of RNAPII-associated transcription factories with the CTCF/cohesin structural foci, and also the prevalently distributed CTCF along the whole chromosomal axis.

The rapidly-increasing attention toward 3D genome-related studies as reflected in recent literature is not an accident. Besides the biological importance *per se*, the advancement in technological innovation has stimulated the blast of the field substantially. It is not hard to predict that the work presented by Ruan and colleagues in the highlighted study will become a new milestone along the ever-growing family of chromatin contact mapping technologies. With the reduced cost of new technologies, 3D genome-related studies become affordable toward more and more laboratories, which will, obviously, expand the field of 3D genome research by exploring more cell types, tissues, and species, thus triggering more research interests in developmental biology, population genetics, evolution, and clinical medicine.

## Competing interests

The author has declared that no competing interests exist.
